# Involvement of the Membrane Nanodomain Protein, *At*Flot1, in Vesicular Transport of Plasma Membrane H^+^-ATPase in *Arabidopsis thaliana* under Salt Stress

**DOI:** 10.3390/ijms24021251

**Published:** 2023-01-08

**Authors:** Lyudmila A. Khalilova, Olga V. Lobreva, Olga I. Nedelyaeva, Igor V. Karpichev, Yurii V. Balnokin

**Affiliations:** K. A. Timiryazev Institute of Plant Physiology, Russian Academy of Sciences, Botanicheskaya Str. 35, 127276 Moscow, Russia

**Keywords:** *Arabidopsis thaliana*, endocytosis, exocytosis, H^+^-ATPase, plasma membrane, salt stress, vesicular transport

## Abstract

The aim of this study was to elucidate whether the membrane nanodomain protein *At*Flot1 is involved in vesicular transport pathways and regulation of the P-type H^+^-ATPase content in plasma membrane of *A. thaliana* under salt stress. Transmission electron microscopy revealed changes in the endosomal system of *A. thaliana* root cells due to knockout mutation SALK_205125C (*Atflot1ko*). Immunoblotting of the plasma membrane-enriched fractions isolated from plant organs with an antibody to the H^+^-ATPase demonstrated changes in the H^+^-ATPase content in plasma membrane in response to the *Atflot1ko* mutation and salt shock. Expression levels of the main H^+^-ATPase isoforms, *PMA1* and *PMA2*, as well as endocytosis activity of root cells determined by endocytic probe FM4-64 uptake assay, were unchanged in the *Atflot1ko* mutant. We have shown that *At*Flot1 participates in regulation of the H^+^-ATPase content in the plasma membrane. We hypothesized that *At*Flot1 is involved in both exocytosis and endocytosis, and, thus, contributes to the maintenance of cell ion homeostasis under salt stress. The lack of a pronounced *Atflot1ko* phenotype under salt stress conditions may be due to the assumed ability of *Atflot1ko* to switch vesicular transport to alternative pathways. Functional redundancy of *At*Flot proteins may play a role in the functioning of these alternative pathways.

## 1. Introduction

Membrane vesicles travel between membrane-bound cellular compartments, transporting various compounds and signaling molecules. Exocytic vesicles carrying newly synthesized molecules, as well as molecules undergoing recycling, move to the cell surface where, by fusion with the plasma membrane (PM), they transfer membrane material into this organelle and release the lumen content into the apoplast. Through endocytic vesicles, PM material and substances from the apoplast are internalized by the cell [1,2,3,4,5,6]. The PM protein composition is regulated by changes in the exocytosis/endocytosis rates occurring in response to internal and external stimuli [1]. The regulation of PM protein abundancies via vesicular trafficking pathways is known for the inward rectifying K^+^ channel KAT1 [7], aquaporin plasma membrane intrinsic protein (PIP2) [8], respiratory burst oxidase homolog protein D (RbohD) [9], boron efflux transporter BOR1 [10], indolyl-3-acetic acid (auxin) carriers PIN1 and PIN2 [11], the iron-regulated transporter 1 (IRT1) [12], and others in *A. thaliana* cells.

Some of these proteins are trafficked through flotillin-dependent pathways. Particularly, the aquaporin PIP2 internalization that reduces PM water permeability when roots are treated with NaCl was shown to occur with the involvement of a flotillin-dependent pathway [8]. Furthermore, RbohD is a protein localized in *A. thaliana* PM nanodomains which contributes to the activation of redox signaling pathways also subjected to flotillin-dependent endocytosis in response to salt stress [9].

Flotillins play multiple roles in mammalian and yeast cells. These proteins are involved in organization of membrane nanodomains, signaling, regulation of ion and water exchange, endocytosis, control of vesicular protein traffic, cholesterol uptake, intercellular communications, cell response to pathogens, and in cancer development [13,14,15]. In plants, however, the flotillins have been studied to a much lesser extent than in animals and yeasts. The functional roles of flotillins in mechanisms responsible for ion homeostasis and salt tolerance in plants have not yet been clarified. At the same time, there is some evidence that may indirectly indicate the involvement of flotillins in plant ionic relations and salt tolerance. Indeed, *A. thaliana* Flot2 has been shown to directly interact with plasma membrane ATPase1 [16], a protein involved in the energization of Na^+^ exports from the cytoplasm [17]. Transcription of flotillin genes in *A. thaliana* was affected by a number of stress factors including NaCl [18]. The *At*Flot2 was binding to CPA2, a monovalent cation transporter, and all three *At*Flot isoforms interacted with cornichon [16], an *A. thaliana* protein of unknown function, an orthologue of which forms a complex with the K^+^/Na^+^ transporter in rice [19]. In an *A. thaliana* mutant with a T-DNA insertion in the *AtFlot1* gene promoter, which is characterized by increased expression of this gene, a higher content of K^+^ and a lower content of Na^+^ were found under salt stress when compared with wild type (WT) plants [20]. This mutant also developed organs with higher mass than WT when plants were grown under salt stress. It is possible that flotillins may be involved in ion homeostasis and processes underlying salt tolerance in plants by regulating in the PM content of the ion-transporting proteins including P-type H^+^-ATPase. The latter plays a key role in PM energization by creating transmembrane gradients of electrical potential and pH, which are energy sources for K^+^ uptake by cells and Na^+^ export from the cytoplasm, respectively [17,21,22].

The aim of this study was to clear up the question as to whether the membrane nanodomain protein *At*Flot1 is involved in the vesicular transport pathways of the P-type H^+^-ATPase in *A. thaliana* under salt stress conditions. For this purpose, we performed a Western blot analysis of P-type H^+^-ATPase on plasma membrane preparations isolated from the roots and leaves of *A. thaliana* WT plants and the *Atflot1ko* knockout mutant for the *AtFlot1* gene, SALK_205125C [23]. In addition, in the organs of these plant lines, we studied H^+^-ATPase expression by real-time qRT-PCR, and in the ultrastructure of root cells with an emphasis on the endosomal system using transmission electron microscopy (TEM), as well as endocytosis activity by analysis of uptake dynamics of an endocytic probe, FM4-64. Studies were performed on plants exposed or not exposed (control) to salt stress. The obtained results testify in favor of the involvement of the *At*Flot1 protein in both exocytosis and endocytosis pathways that regulate the P-type H^+^-ATPase in *A. thaliana* root cells.

## 2. Results

### 2.1. Characterization of SALK_205125C Mutant Plants

**Figure 1:**
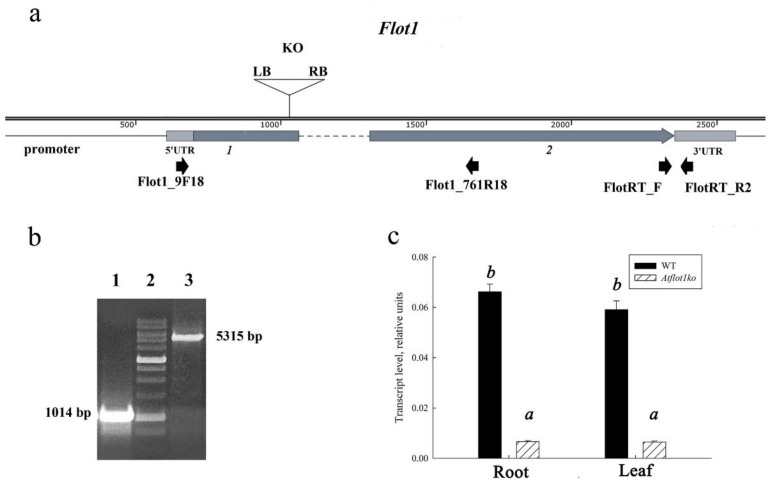
Structure of the *AtFlot1ko* allele of the *A. thaliana AtFlot1* gene. (**a**) Arrangement of the functional elements in the *AtFlot1* gene. Here, *1* and *2* indicate exons and untranslated regions. Intron sequence is indicated with the broken line. The triangle indicates the T-DNA insert in the *Atflot1ko* (KO) allele. Arrows in the bottom line indicate positions of primers used. (**b**) Determination of homozygosity for the *AtFlot1* gene in the SALK_205125C mutant plants by PCR using a pair of primers, namely Flot1_9F18 and Flot1_761R. The amplification products were separated on an 1% non-denaturing agarose gel containing 0.5 μg/mL ethidium bromide; 1—WT, 2—molecular weight ladder, 3—*Atflot1ko*. (**c**) Expression of the *AtFlot1* gene in the wild-type (WT) and *AtFlot1ko A. thaliana* mutant plants. Means ± SE are given (*n* = 3). Different letters indicate statistically significant differences between the samples (ANOVA; *p* < 0.05).

In the current study, we used the SALK_205125C *AtFlot1* knockout mutant. According to the data provided in the Arabidopsis Information Resource (TAIR) database, the T-DNA insert in the SALK_205125C mutant is located at position −10 bp from the start codon of the *AtFlot1* gene, and the T-DNA left border sequence (LB) in this mutant is located in forward direction in relation to nucleotide numbering in the chromosome (Figure 1a). We confirmed the presence of a T-DNA insert in the SALK_205125C mutant by PCR amplification using a mutant genomic DNA template with a pair of primers Flot1_9F18 and Flot1_761R18 (Table 1) and obtained a predicted amplicon of ≈5300 bp in size.

To determine exact coordinates of the insert location in the *AtFlot1* gene, the obtained PCR fragment was sequenced using the Flot1_9F18 primer (Table 1). We have found that the T-DNA insert in the SALK_205125C mutant is located at the end of the first exon, 321 bp downstream from the start codon, and that the T-DNA LB sequence direction is opposite to the nucleotide numbering direction of the chromosome (Figure 1a). We also confirmed that the SALK_205125C *AtFlot1* mutant line is indeed a knockout mutant by analyzing the expression of the *AtFlot1* gene in these mutant plants (Figure 1c). We only observed the “residual” expression of *AtFlot1* at extremely low levels in this mutant when compared to WT levels. Finally, we have determined whether the SALK_205125C mutant plants were homozygous for *AtFlot1* T-DNA knockout allele by PCR using a pair of primers, namely Flot1_9F18 and Flot1_761R. The primers were designed in such a way that in the presence of the wild type allele, a fragment of 1014 bp would have been amplified, whereas in the presence of the T-DNA insert, the amplicon of 5315 bp would have been produced. We have confirmed that these plants were indeed homozygous for the mutant *AtFlot1* allele (Figure 1b).

It is also worth noting that re-analysis of the *Atflot1* sequencing data indicated that the T-DNA insert in *Atflot1oe* mutant line used by us earlier [20] is located at position −91 bp rather than at position −41 bp from the transcription start site, and the last 4 nucleotides 5′GCGC3′ of the left T-DNA border (LB) sequence have been replaced by a 10-nucleotide sequence 5′ATTGGTAATC3′.

Inverse PCR was the method of choice to determine possible presence of additional T-DNA inserts in the genome of the *Atflot1ko* mutant. The PCR amplification, using genomic DNA digested with various restriction enzymes and then ligated as a template, yielded specific amplicons with predicted molecular masses corresponding to the *AtFlot1* gene only in the case of DNA obtained from mutant plants. These amplicons were then sequenced to verify their identities. Specific amplification products other than those predicted were not observed, which allows us to conclude that, most likely, the *Atflot1ko* mutant does not contain additional T-DNA inserts.

**Figure 2:**
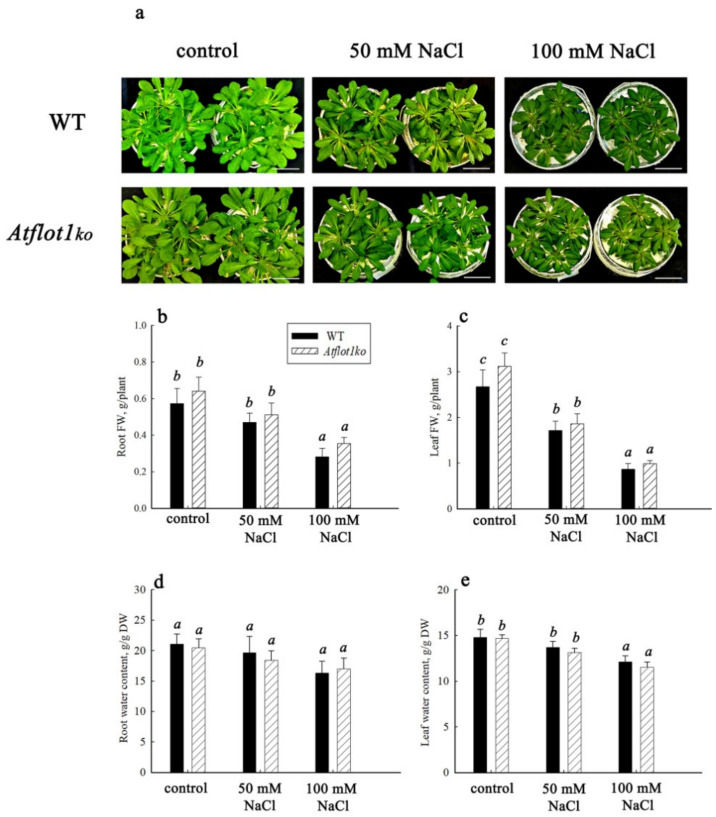
Growth characteristics of *A. thaliana* WT and *Atflot1ko* mutant plants and hydration levels of the plant organs. The plants were grown either in the absence (control) or presence of NaCl in the nutrient solution (NS) at final concentrations of 50 or 100 mM; (**a**) general view of plants, scale bar—5 cm; (**b**,**c**) fresh weight (FW) of roots and leaves; (**d**,**e**) water content in roots and leaves. Means ± SE are given (*n* = 3, average weight from 16 plants in each of the repetition). Different letters indicate statistically significant differences between the samples (ANOVA; *p* < 0.05).

To characterize the phenotype of the *Atflot1ko* knockout mutant (Figure 2a), we studied growth, hydration level, and the content of Na^+^ and K^+^ in organs of plants grown either in the absence of NaCl or under salinity conditions. In *Atflot1ko*, inhibition of plant growth was not seen (Figure 2b,c). The fresh weight (FW) of roots and leaves of 45-day-old *Atflot1ko* plants grown in aquatic culture both in the absence (control) and presence of NaCl, was similar to the FW found for WT plants (Figure 2b,c). The tissue hydration levels under these conditions were similar in *Atflot1ko* and WT plants (Figure 2d,e), which generally indicates the absence of differences in dry weight as well and the lack of inhibition of biosynthetic processes when the *Atflot1* gene was knocked out.

**Figure 3:**
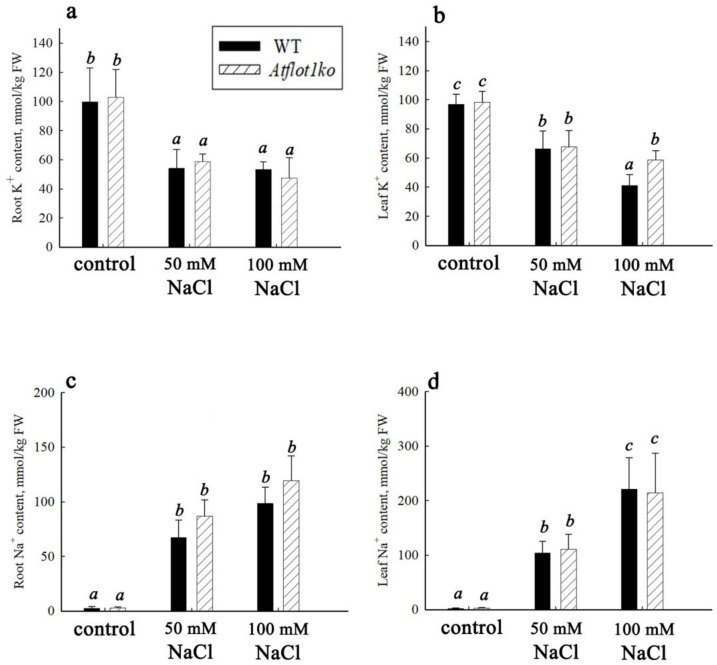
K^+^ (**a**,**b**) and Na^+^ (**c**,**d**) content in roots and leaves of *A. thaliana* WT and *Atflot1ko* knockout mutant plants grown either in the absence (control) or presence of NaCl in the medium at final concentrations of 50 and 100 mM. Here, (**a**,**c**) roots, and (**b**,**d**) leaves. Means ± SE are given (*n* = 15). Different letters indicate statistically significant differences between the samples (ANOVA; *p* < 0.05).

Under these conditions, no statistically significant differences were found between WT and *Atflot1ko* plants in the content of K^+^ (Figure 3a,b) and Na^+^ (Figure 3c,d). This result indicates that the knockout mutant plants are able to maintain Na^+^ and K^+^ homeostasis, even under salinity conditions.

### 2.2. Immunoblot Analysis of Plasma Membrane H^+^-ATPase in Atflot1ko Mutants

To demonstrate the effects of the mutation and salinity stress on the P-type H^+^-ATPase abundance in PM of *A. thaliana* cells of WT and *Atflot1ko*, we determined the relative content of this protein in plasma membranes isolated from roots and leaves of WT and *Atflot1ko* plants grown in the aquatic culture conditions in the absence of NaCl in NS and then subjected or not subjected (control) to salt shock (100 mM NaCl, 12 h). We chose P-type H^+^-ATPase as a protein that plays a key role in ion homeostasis, especially under salinity condition.

**Figure 4:**
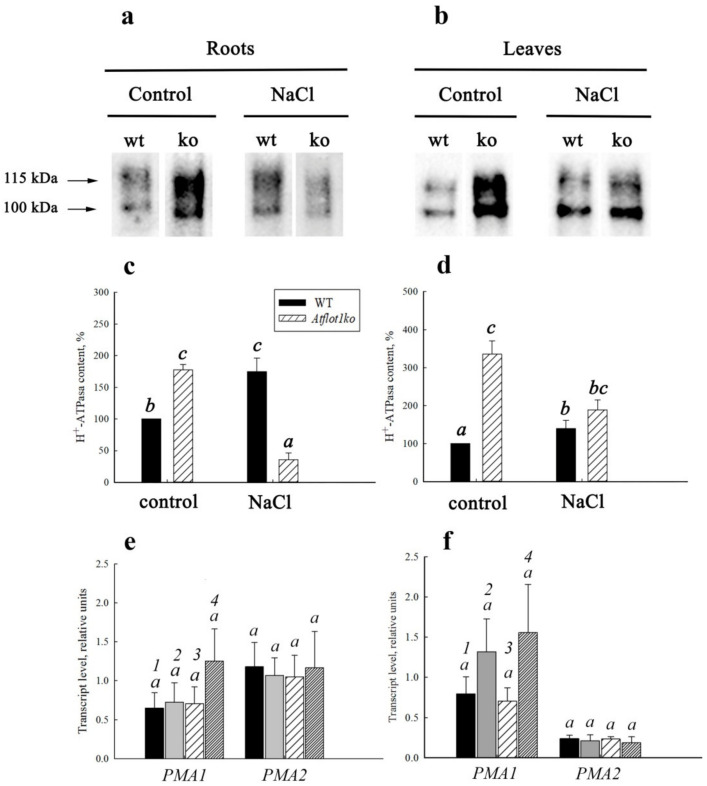
The effect of *Atflot1ko* mutation and salt shock on the content of H^+^-ATPase in PM membrane preparations from organs of *A. thaliana* plants and on the gene expression of two isoforms of this protein, namely *PMA1* and *PMA2*. (**a**,**b**) Immunoblotting of membrane preparations obtained from roots (**a**) and leaves (**b**) of *A. thaliana* WT and *Atflot1ko* (ko) plants subjected or not subjected (control) to salt shock. (**c**,**d**) Quantitative analysis of the content of H^+^-ATPase isoforms in root cell PM of WT and *Atflot1ko* under control or salt shock conditions. The value for WT under control conditions was taken as 100%. Means ± SE are given (*n* = 3). (**e**,**f**)—The expression of the *PMA1* and *PMA2* genes in roots (**e**) and leaves (**f**) *of A. thaliana* WT and *Atflot1ko* plant lines under control or salt shock conditions. Means ± SE are given (*n* = 3). Here, *1*—WT, control, *2*—WT, 100 mM NaCl, *3*—*Atflot1ko*, control, and *4*—*Atflot1ko*, 100 mM NaCl. Different letters indicate statistically significant differences between samples (one-way ANOVA; *p* < 0.05).

The P-type H^+^-ATPase on the Western blot lanes appeared as two immunoreactive bands with close molecular masses in the range of 100–115 kDa (Figure 4a,b), which, apparently, reflects the interaction of antibodies with different H^+^-ATPase isoforms. Two similar immunoreactive P-type H^+^-ATPase bands were detected in *A. thaliana* by the others [24].

Under control conditions, in PM isolated from both roots and leaves of the *Atflot1ko* plants, the relative content of H^+^-ATPase per weight unit in the membrane fraction was noticeably higher than in WT. Salt shock also led to an increase in H^+^-ATPase content in PMs isolated from the organs of WT plants. However, the increase in H^+^-ATPase content in PM under the combined action of both the mutation and salt shock was smaller when compared with effect of only the mutation, and even the H^+^-ATPase content in PM became smaller than in WT in control conditions (Figure 4c,d).

### 2.3. Expression of Plasma Membrane H^+^-ATPase Genes in Atflot1ko Mutant

To evaluate the contribution of the changes in H^+^-ATPase expression levels caused by *Atflot1ko* mutation or NaCl shock to the H^+^-ATPase content in PM, we studied the expression of two main H^+^-ATPase isoforms, namely *PMA1* (At2g18960) and *PMA2* (At4g30190) [24] in roots and leaves of these plants under control and NaCl shock conditions (Figure 4e,f). The WT and *Atflot1ko* plants showed no statistically significant differences in the relative levels of transcripts of the H^+^-ATPase isoforms in roots and leaves, both in control and under salt shock conditions (Figure 4e,f). In general, it may be concluded that the effect of knockout mutation on H^+^-ATPase relative content in *A. thaliana* root cell PM is mediated rather by changes in vesicular trafficking than by alterations in expression levels of the H^+^-ATPase isoforms. The NaCl shock demonstrated a tendency to stimulate *PMA1* expression in leaves of WT and *Atflot1ko* and in roots WT as well (Figure 4e,f).

### 2.4. Endocytic Probe FM4-64 Uptake by Roots of Atflot1ko Mutant Seedlings

**Figure 5:**
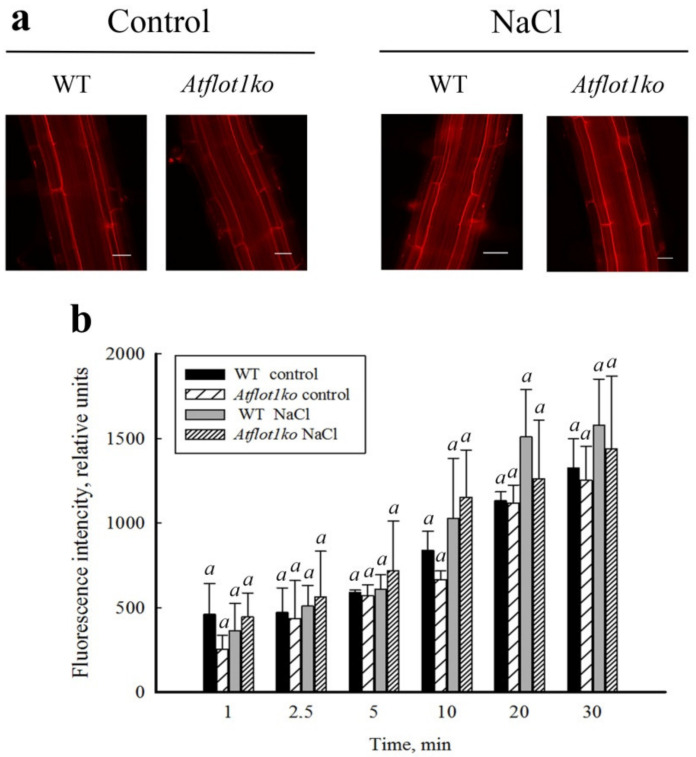
Dynamics of the fluorescent marker FM4-64 uptake by roots of 5-day-old *A. thaliana* seedlings incubated in ½ MS medium containing 2 µM of FM4-64, either in the absence or presence of NaCl. (**a**) Color images show the intensity of FM4-64 fluorescence in root cells 30 min after incubation in a dye solution. (**b**) A time course of FM4-64 uptake by roots of 5-day-old *A. thaliana* seedlings. Means ± SE of FM4-64 fluorescence intensity in roots in 3 independent experiments using 50 roots in every experiment are given. Scale—20 µm. Identical letters indicate no difference between mean values in each group (one-way ANOVA, *p* < 0.05).

To evaluate effects of *Atflot1ko* mutations and salt shock stress on the endocytic activity of *A. thaliana* root cells, we studied the uptake dynamics of FM4-64, a fluorescent marker of endocytic structures, by roots in 5-day-old seedlings of WT and *Atflot1ko* plants subjected or not subjected to salt shock. The seedlings of WT and *Atflot1ko* plants not subjected to salt shock absorbed PM fluorescent dye almost at equal rates, and noticeable changes in dye absorption rates in response to NaCl shock in seedlings of the both lines were not observed either (Figure 5a,b). This result suggests that endocytosis does not contribute to *Atflot1*- and NaCl shock-mediated changes in the abundance of P-type H^+^-ATPase in PM.

### 2.5. Ultrastructural Peculiarities of Root Cells in the Knockout Mutant Atflot1ko Plants

**Figure 6:**
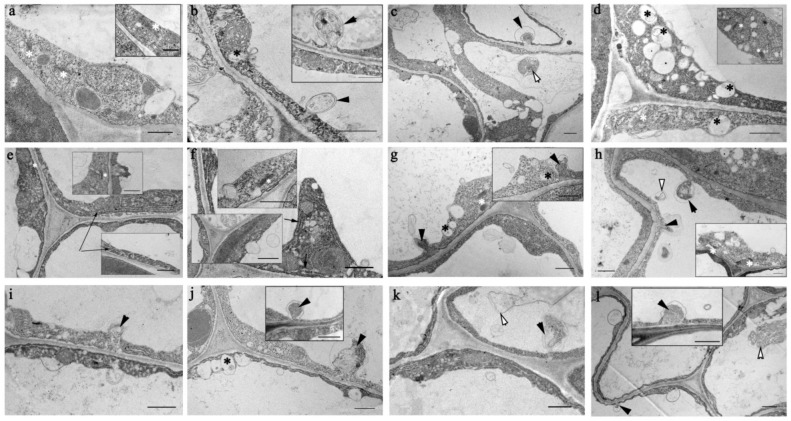
Transmission electron micrographs of root cells of 4-day-old seedlings of *A. thaliana* WT (**a**–**d**) and *Atflot1ko* knockout mutant (**e**–**l**) grown either in the absence (**a**,**b**,**e**–**h**) or in the presence (**c**,**d**,**i**–**l**) of NaCl. Black asterisks indicate multivesicular bodies (MVBs), white asterisks indicate Golgi complexes (GCs), black short arrows indicate paramural bodies (PMBs), white short arrows indicate derivatives of paramural bodies (PMBDs), and black long arrow indicate the ER. Scale—1 µm.

Using TEM, we studied ultrastructure of the *A. thaliana* WT and *Atflot1ko* root cells, focusing on the Golgi complex (GC) and endosomal system. The plants were grown either in the presence or absence (control) of NaCl in the medium. Under control conditions, the Golgi apparatus was active in both WT (Figure 6a,a-insert) and *Atflot1ko* (Figure 6e,e-insert,f,f-top insert) plants; this was indicated by vesicles budding from the dictyosomes and trans-Golgi network/early endosomes (TGN/EE). Membranes of the endoplasmic reticulum (ER) in the knockout mutant plants often diverged, forming gaps and cavities that are not characteristic of the ER of WT plants grown in the absence of NaCl in the medium (Figure 6e,e-insert,f). Vesicles budding into the cytoplasm, both in WT and in the knockout mutant, were of different sizes, in the range of approximately 30–250 nm. The vesiculation in *Atflot1ko* appeared to be more active than in WT. In some knockout mutant cells, vesicles occupied a significant part of the cytoplasmic volume (Figure 6e,e-insert,f,f-bottom insert).

**Figure 7:**
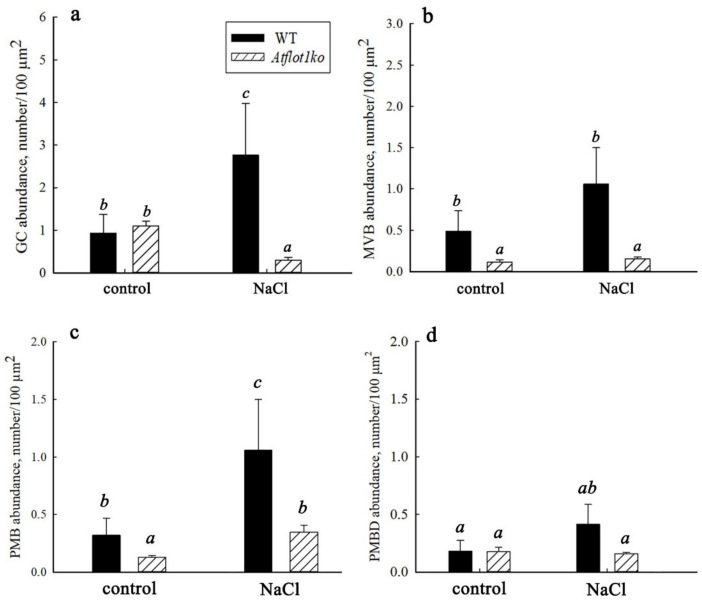
The frequency of occurrence of GCs (**a**), MVBs (**b**), PMBs (**c**), and PMBDs (**d**) in the root cells of 4-day seedlings of *A. thaliana* WT and *Atflot1ko* plants grown either in the absence or in the presence of NaCl. Means ± SE using 100 individual cells are given (*n* = 100). Different letters indicate statistically significant differences (ANOVA; *p* < 0.05).

**Figure 8:**
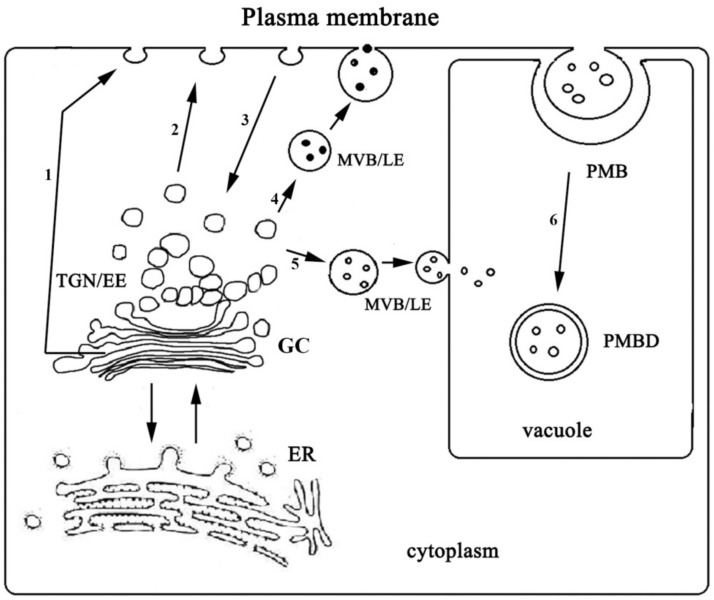
A hypothetical scheme outlining effects of *Atflot1ko* mutation on the vesicle trafficking pathways and P-type H^+^-ATPase content in the plasma membrane of *A. thaliana* root cells in control and salt stress conditions. Abbreviations are as follows: ER—endoplasmic reticulum, TGN/EEs—trans-Golgi network/early endosomes, MVBs/LEs—multivesicular bodies/late endosomes, PMB—paramural body, and PMBD—derivative of paramural body. Vesicular trafficking pathways are as follows: 1, 2, 4—exocytosis; 3, 6—endocytosis, 5—vacuolar pathway. Under control conditions, the inhibition of the canonic pathways proceeding via LE/MVB formation (4, 5), stimulation of alternative exocytosis pathways bypassing the formation of MVBs (1, 2), and increase in P-type H^+^-ATPase content in PM occur. Under salt stress conditions, the inhibition of GC production resulting in elimination of both canonic (4, 5) and alternative (1, 2) exocytosis pathways, and a decrease in P-type H^+^-ATPase content in PM, take place. The endocytic MVB-mediated pathway accompanied by the production of PMBDs (6) undergoes alterations by the *Atflot1* mutation and NaCl stress.

Under control conditions, the contents of GCs per unit section area in *Atflot1ko* and WT plants were almost equal (Figure 7a), while the MVB content in the knockout mutant was significantly lower than in WT (Figure 7b). We also determined the content of paramural bodies (PMBs) in the cells. The PMBs are formed by invaginations of two membranes, PM and tonoplast, into the vacuole at once (Figure 6b,b-insert,g,h). When detached from the PM and tonoplast, such invaginations form two-membrane structures in vacuoles which, in this paper, we will refer to as derivatives of paramural bodies (PMBDs) (Figure 6c,h and Figure 8). In the *Atflot1ko* knockout mutant, under these conditions, the PMB content was lower than in WT (Figure 7c).

When plants were grown on a NaCl-containing medium (100 mM), the ultrastructure of root cells underwent changes. We have found increased vacuolization and vesiculation of the cytoplasm (Figure 6c,d), fusion of small vacuoles into larger ones, an increase in the number of GCs, more frequent budding of vesicles from secretory pole of GCs, and the formation of more MVBs than in the control (Figure 7a,b) in plants grown on NaCl-containing media. Adding NaCl to the media led to a noticeable increase in the content of PMBs in WT plants (Figure 7c). The knockout mutation on the background of salinity caused a significant decrease in the content of GCs and MVBs in the root cells (Figure 7a,b). In knockout mutant plants, under salt stress conditions, the content of PMBs was lower than that in WT and maintained at the WT levels under control conditions (Figure 7c). The opposite effect of the knockout mutation on the content of GCs, MVBs, and PMBs in relation to the effect of NaCl should be noted. In the knockout mutant, the PMBD content under both conditions remained at the levels seen in WT under control conditions (Figure 7d).

The effects of NaCl on the H^+^-ATPase content in PM, endocytosis activity, and content of the membrane-bound intracellular structures in the cells of WT and *Atflot1ko* are summarized in Table 2.

## 3. Discussion

One of the ways to regulate the abundance of ion-transporting proteins in the plasma membrane is to make alterations in the ratio of exocytosis and endocytosis rates, which is a key regulatory step in maintaining cell ion homeostasis [25]. To implement such regulation, plants evolved sophisticated system acting in all steps of the trafficking pathways [26,27,28,29,30,31,32,33]. Increase under control conditions in H^+^-ATPase content in the PM of root and leaf cells as a result of the knockout mutation and weakening of the mutation′s effect under salt shock conditions observed in the immunoblotting assay (Figure 4a,b) could be explained by alterations in exocytosis/endocytosis rates ratio. Apparently, in the absence of NaCl, the rate of protein delivery to PM (exocytosis) exceeded the rate of protein transfer from PM to cytoplasm (endocytosis). Sodium chloride added to the medium reversed the situation in *Atflot1ko*. The delivery rate of the protein to PM apparently became lower than the rate of its endocytosis (Table 2). A contribution to the decrease in the content of H^+^-ATPase in PM was probably also made by the vacuolar pathway of protein degradation. The increased H^+^-ATPase content in PM under salt shock in WT, which distinguishes it from the knockout mutant, probably results from the excess of the exocytosis rate over the endocytosis rate occurring under these conditions.

We compared the effect of the *Atflot1ko* mutation on the relative H^+^-ATPase content in PM (Figure 4a–d) with the effect of this mutation on the ultrastructural characteristics (Figure 6, Figure 7 and Figure 8) and endocytic activity that was studied by the FM4-64 uptake assay of roots (Figure 5). Large numbers of the vesicles, derived supposedly from the ER, GC, or EE, and a low content of MVBs in cytoplasm of knockout mutant cells under control conditions (Figure 6e,f and Figure 7b) may indicate suppression of MVBs/LEs formation (Figure 8, pathways 4, 5) and activation of direct cargo transport by the vesicles from GCs or EEs to PM (Figure 8, pathways 1, 2) bypassing the canonical pathway through MVB/LE formation. The vesicular transport pathways, which bypass the MVB formation, have been shown in a number of studies [4,5,6,34,35,36]. It is also possible that the low content of MVBs in the knockout mutant grown under control conditions may be due to an increased rate of MVB fusion with PM, which leads to accelerated delivery of cargo to PM and, accordingly, to a faster consumption of the MVB membrane material. However, a very rare occurrence of exosomes in periplasmic space in the knockout mutant cells under control conditions (Figure 6e–h) is rather in line with the inhibition of formation of MVBs, cytoplasmic membrane structures that deliver exosomes to periplasm. The low content of GCs along with low content of MVBs observed in the knockout mutant under salt stress conditions (Figure 7a,b) testify in favor of exocytic pathway inhibition and the involvement of the *At*Flot1 protein in exocytosis on the stage of Golgi complex formation. In general, it may be assumed that *At*Flot1 is required for operation of the canonical MVB-dependent secretory pathway, which is active in WT under control and salt shock conditions.

It is likely that a lack of the *At*Flot1 protein in the knockout mutant cells makes the canonic exocytic pathway sensitive to NaCl. In WT plants not subjected to salt shock, the content of H^+^-ATPase in PM corresponds to a balanced state of exocytosis and endocytosis. This state is probably determined by a physiological range of *At*Flot1 content in cells, which is a natural characteristic of WT plants. Within this range, NaCl shock, apparently through stimulating the MVB-mediated pathway, increases the content of H^+^-ATPase in PM of root and leaf cells in WT plants (Figure 4a,b; Table 2). According to our hypothesis, in *Atflot1ko*, NaCl shock leads to inhibition of the exocytosis pathway. The assumed predominance of endocytosis over exocytosis under these conditions in *Atflot1ko* is possibly also due to the accelerated endocytosis. However, the study of the uptake dynamics of the endocytic probe FM4-64 by roots of WT and *Atflot1ko* seedlings did not reveal any noticeable differences between the two plant lines in rates of the probe uptake, both under control (Figure 5a) and under salt stress conditions (Figure 5b), testifying rather in favor of alteration in the H^+^-ATPase PM content through changes in exocytosis rate.

Plasma membrane P-type H^+^-ATPase is the generator of transmembrane proton motive force (pmf), both components of which, namely proton gradient (ΔpH) and electric potential (Δφ), are energy sources for nutrient absorption and maintaining ion homeostasis in plant cells [37]. Indeed, ΔpH plays an important role in plants under salinity stress by energizing Na^+^ exported from the cytoplasm by Na^+^/H^+^ antiporters [17]. However, the other component of pmf, Δφ, along with the energization of uptake of the substances required for the cell, is a motive force for entering the cell of toxic Na^+^ ions [21]. Therefore, changes in the H^+^-ATPase abundance in PM by alterations in exocytosis/endocytosis ratio for this protein could be the means for rapid regulation of Na^+^ entering the cells under salinity oscillations.

One of the pathways that contributes to the transport of molecules into vacuoles is forming PMBs, with consequent transformation of PMBs into PMBDs. Most likely, these structures are involved in the transfer of not only PM and tonoplast proteins into the vacuolar lumen, but also the cytoplasmic material located between these membranes, as well as substances contained in apoplast (Figure 8).

The content of PMBs, the structures retaining a connection with cytoplasm, should depend both on the rate of their formation and on the rate of their separation from cytoplasm and transition to a free state in the vacuole. The NaCl in the medium noticeably stimulated PMB and PMBD formation in WT and *Atflot1ko* root cells. Reduced contents of PMBs in both the absence and presence of NaCl in the medium and PMBDs under salinity in *Atflot1ko*, when compared to WT plants conditions (Figure 7c,d), suggest that *At*Flot1 may be involved in both PMB formation and in subsequent PMB transformation into PMBDs.

In general, the results outlined above as well obtained previously [20] testify in favor of the involvement of *At*Flot1, the membrane nanodomain protein, in various pathways of vesicular transport in *A. thaliana*, including exocytosis, endocytosis, and the pathway that delivers the substances into the vacuole through PMB biogenesis and the subsequent formation of PMBDs. It is most likely that *At*Flot1 is simultaneously required for several stages of each transport pathway, which is consistent with the idea that flotillins play structural rather than functional roles. Flotillins have not been found to possess activities similar to those of enzymes or transporters. The role of these proteins is probably to affect other proteins through protein–protein interactions [38]. It has been shown that *At*Flot2 forms complexes with proteins involved in various metabolic processes and physiological functions, including ion homeostasis, water transport, intracellular substance trafficking, plant response to biotic and abiotic stresses, ubiquitination, and proteasomal degradation of proteins, among others [16,39]. The proteins interacting with *At*Flot2 also include plasma membrane H^+^-ATPase [16]. Flotillins, taking part in ion and water exchange, may play an important role in the salt tolerance in plants. The relation of salt tolerance to vesicular transport has been shown in a number of studies [8,40,41,42,43,44]. Although flotillins play a role in many physiological processes, we did not find growth suppression (Figure 2) as well as disturbances in Na^+^, K^+^ homeostasis (Figure 3) in the root and leaf cells of the *Atflot1ko* knockout mutant.

This may be due to the activation of alternative vesicular transport pathways, as well as to the execution of *At*Flot1 functions by other flotillin isoforms. The latter, in particular *At*Flot2, may be involved in providing alternative pathways of vesicular transport. The assumption regarding the redundancy of the functions of the individual flotillins *At*Flot1 and *At*Flot2 is supported by the results of the study [45], in which it was shown that an amiRNA line *A. thaliana* plants with reduced expression of both *At*Flot1 and *At*Flot2 were smaller in size than WT plants and showed structural defects in the apical meristem. In addition, Kroumanova et al. [18] found that T-DNA insertion single knockout mutants of *A. thaliana*, which do not transcribe only one individual *AtFlot* gene, when under salt and other abiotic stresses, did not have significant differences in root length and did not show other noticeable morphological changes when compared to WT plants with a parental background phenotype.

At the same time, as shown by the results of our previous work [20], elevated expression of *AtFlot1* in root cells of the *Atflot1oe* mutant led to a noticeable stimulation of growth, an increase in K^+^ content, and a decrease in Na^+^ content in the organs of this mutant during salinity stress, indicating the importance of the *At*Flot1 protein for the proper growth of *A. thaliana* and maintenance of ion homeostasis in its cells under these conditions. It is well known that the phenotype observed upon overexpression of a target protein may reflect the authentic biological role played by this protein [46] and, thus, the involvement of the *At*Flot1 protein in salt tolerance is supported by previous and current findings.

In summary, the phenotypes of *Atflot1* mutant plant lines described in this study and earlier [20] under salt stress conditions, together with the results obtained by the other groups [16,18,39], suggest that none of the *At*Flot isoforms play an exclusive role in the adaptation of *A. thaliana* to all tested abiotic stresses, including salinity. The explanation may be the multiplicity of vesicular protein transport pathways and the ability of the *Atflot1ko* knockout mutant to switch to alternative pathways that ensure proper delivery of proteins to the respective membranes. The functional redundancy of proteins in the *At*Flot family may play an important role in the functioning of alternative pathways, in which other flotillin isoforms perform *At*Flot1’s function.

## 4. Material and Methods

### 4.1. Plant Material

This study was carried out using *Arabidopsis thaliana* (L.) Heynh., Col-0 ecotype wild type (WT) plants and a knockout mutant plant for the *AtFlot1* gene *Atflot1ko* (SALK_205125C). All experiments, except for cell ultrastructure studies and the endocytic probe FM4-64 uptake assays, were performed on plants grown under aquatic culture conditions. The seeds were germinated on agarized medium prepared with modified ⅓ Hoagland nutrient solution (http://scienceinhydroponics.com/2009/02/the-hoaglands-solution-for-hydroponic-cultivation.html, accessed on 14 November 2019), at 23 ± 2 °C, relative air humidity of 70 ± 5%, photoperiod of 12 h per day, and light intensity of 120 µmol quanta/m^2^·s emitted from Phillips TL-D-58W/33-640 fluorescent lamps (Poland). Here, 15-day-old-seedlings were transferred on aerated ½ Hoagland nutrient solution (NS) in 1 L plastic jars (4 plants in each jar). Other conditions for growing plants on NS were identical to those described for seed germination. Measurements of the weight of organs, determination of the ion content, and the gene expression studies (*AtFlot1*, *PMA1*, *PMA2*) were carried out on 45-day-old plants. In the variants with salinization, 10 days after the transfer of seedlings on liquid media, NaCl was added to the media to final concentrations of either 50 or 100 mM. The membrane fractions enriched with PM for immunoblot analysis of the P-type H^+^-ATPase were isolated from the roots of 50-day-old plants. In the variants with salt shock, NaCl was added to the NS 12 h prior to the membrane isolation. The nutrient solutions were changed on a weekly basis.

To study root cells ultrastructure, 4-day-old seedlings were used. The seedlings were grown on ½ Murashige–Skoog (½ MS) agarized nutrient medium containing 0.5% sucrose, either with or without addition of 100 mM NaCl.

To study absorption of the endocytic probe FM4-64 by roots, seedlings were grown on the same medium containing 0.5% sucrose until 5 days of age in the absence of NaCl. The temperature and light conditions in these experiments were identical to those described for growing plants in aquatic culture.

### 4.2. Isolation of Plant Genomic DNA

Genomic DNA from *A. thaliana* leaves was isolated using a CTAB method described previously [47] with some modifications, as follows. A total of 200 mg of plant tissue in a 1.5 mL test tube was ground with Celite and 200 µL of MicroPrep lysis buffer (0.088 M Sorbitol, 0.075 M Tris, 0.014 M EDTA, 0.5 M NaCl, 0.5% CTAB, and 0.5% Sarkosyl, pH 7.5) containing 0.1% sodium bisulfite (added just before use); then, 550 μL more buffer was added to the slurry. Tubes were heated at 65 °C for 1 h, 700 µL of chloroform was then added, and samples were vortexed and spun for 5 min at 12,000× *g* at +4 °C. Supernatants were recovered and transferred into fresh tubes, and 700 µL of isopropanol was added. Tubes were inverted, incubated 3–5 min at RT until DNA precipitated, spun for 5 min at 12,000 at room temperature (RT), and then the supernatants were discarded. Five hundred µL of 70% ethanol was added to the pellet, the tubes were mixed by inversion, and then they were centrifuged again for 5 min at 12,000× *g* at RT. The DNA precipitates were dried on air for 10–15 min and dissolved in 200 µL sterile milliQ water. One µL of DNase-free RNase solution (10 mg/mL) was added, and tubes were incubated at 37 °C for 30 min to digest the RNA. The obtained DNA preparations were stored at +4 °C. The amplified PCR fragments using purified genomic DNA as a template were sequenced, if necessary, at Evrogen (Moscow, Russia).

### 4.3. Determination of Homozygosity of SALK_205125C Mutants

Genomic DNA purified from *A. thaliana* SALK_205125C plants was used as a template for determining homozygosity by PCR. For this purpose, a pair of primers, namely Flot1_9F18 and Flot1_761R1, was designed (Table 1). The program for PCR was chosen as follows: 1 cycle—94 °C, 2 min; 33 cycles—92 °C, 30 s; 59 °C, 20 s; 70 °C, 3 min; cycle—70 °C, 10 min. The amplification products were analyzed on an 1% non-denaturing agarose gel containing 0.5 μg/mL ethidium bromide. All further experiments were carried out using homozygous mutant plant lines.

### 4.4. Quantitative Real-Time PCR (qRT-PCR)

Isolation of total RNA from *A. thaliana* and synthesis of the first cDNA strand was carried out as described earlier [42]. Here, qRT-PCR was used to analyze the expression of the *PMA1* (At2g18960) and *PMA2* (At4g30190) genes in mutant and WT plants. The gene sequences were taken from the http://www.arabidopsis.org database (accessed on 1 July 2020). Primers for qRT-PCR were selected using the Primer-BLAST program (https://www.ncbi.nlm.nih.gov/tools/primer-blast/, accessed on 15 July 2020). The amplicon size when using the selected pair of primers, AtPMA1_F1 and AtPMA1_R1, (Table 1) for the *PMA1* gene was 111 bp; for the *PMA2* gene using the AtPMA1_F1 and AtPMA1_R1 pair (Table 1) it was 110 bp; for the *AtFlot1* gene with the pair of primers FlotRT _F and FlotRT_R2, it was 109 bp. The *ACT2* gene (NM_001338358.1) was used as a reference gene, and the primer sequences for this gene that were published earlier [20] are listed in Table 1.

The amplification program was created in accordance with the manufacturer recommendations for the Light Cycler^®^ 96 instrument (Roche, Switzerland) and the instructions for the Ready Mix for PCR qPCRmix-HS SYBRmix kit (Evrogen, Moscow, Russia); the same kit was used to prepare the reaction cocktail. Based on the amount of total RNA taken for the synthesis of the first cDNA strand, 30–100 ng of the template was added to the sample. The qRT-PCR data were analyzed using the Light Cycler^®^ 96 SW 1.1 software.

### 4.5. FM4-64 Endocytic Probe Uptake Assay

The five-day-old seedlings of *A. thaliana* WT and *Atflot1ko* mutant were immersed in a ½ MS liquid medium (pH 5.8) and incubated in this medium at 21 ± 2 °C. At the zero time point, a fluorescent marker of endocytic structures, lipophilic styryl dye N-(3-triethylammoniumpropyl)-4-(6-(4-(diethylamino)phenyl)hexatrienyl) pyridinium dibromide (FM™4-64; Molecular Probes, Eugene, OR, USA) [30] and NaCl at final concentrations of 2 μM and 100 mM, respectively, were added to the incubation medium, or 2 μM FM4-64 alone was added in case of the control. After 2, 5, 10, 15, 20, 25, and 30 min of incubation in the indicated media, the seedlings were picked up and were rinsed with the incubation media free of the probe. The intensity of FM4-64 fluorescence absorbed by the roots was then determined by means of an epifluorescence microscope AxioImager Z2 equipped with an EC Plan-Neofluar × 40/0.75 M27 objective and an AxioCamMRm monochrome digital camera, using AxioVision 4.8 software (all from Carl Zeiss, Jena, Germany). Red fluorescence of FM4-64 was detected with a filter set 14 (λ_ex_ 510–560 nm, λ_em_ > 590 nm). The fluorescence intensity of the probe absorbed by the root elongation zone in each image was assessed using the ZEN Blue program (Carl Zeiss, Germany). The experiment was carried out in 3 repetitions, and for each repetition at least 50 images were obtained in every variant of the experiment. Statistical data processing was carried out using Microsoft Excel 2007.

### 4.6. Growth Characteristics, Determination of Water, Na^+^, and K^+^ Content in Plant Organs

Fresh weight (FW) and dry weight (DW) of plant roots and leaves were determined by the gravimetric method. Hydration levels of the organs were calculated as the differences between FW and DW divided by DW. The organs were dried at 90 °C. For determination of Na^+^ and K^+^ content in roots and leaves, dried plant material was ground into powders and resuspended in distilled water. Ions were extracted by boiling the suspensions for 2 min, and the resulting extracts were filtered. The Na^+^ and K^+^ concentrations in the filtrates were determined using a FPA-2-01 flame photometer (OAO ZOMZ, Sergiev Posad, Russia).

### 4.7. Ultrastructure Studies of A. thaliana Seedling Root Cells

The ultrastructure studies of the root epidermis and cortex cells of *A. thaliana* seedlings of WT and knockout mutant *Atflot1ko*, grown either in the presence or absence of NaCl on an agarized nutrient medium ½ MS containing 0.5% sucrose, were conducted using TEM. Samples were prepared as described previously [48]. Root segments of 2–3 mm in size were cut from roots of seedlings at the distance of 1–2 mm from the root tip. The samples were transferred into 2.5% glutaraldehyde (GA) (*v*/*v*) fixing solution, incubated overnight at +4 °C and additionally post-fixed in 1% (*v*/*v*) OsO_4_ for 3 h at +4 °C. Fixing solutions were prepared in 0.05 M cacodylate buffer, pH 7.0. The fixed plant material was dehydrated in ethanol solutions of increasing concentration from 30% to 96% by volume and then in a mixture of 100% ethanol and 100% acetone (1:1). The dehydrated plant material was sequentially passed through a series of epoxy resin/100% acetone mixtures (Epoxy Embedding Medium, Fluka, cat. no. 2920114) with a gradually increasing proportion of resin. The polymerization of the samples was carried out at 37 °C for a day and then at 56 °C for two days. Ultrathin sections were obtained using an ultra-microtome (C. Reichert Om U3, Vienna, Austria). Sections were placed on grids, treated with uranyl acetate, and viewed in a JEM-1400 TEM (JEOL Ltd., Tokyo, Japan). To obtain morphometric parameters, the AxioVision Rel software. 4.8, “Outline” tool was used (Carl Zeiss, Oberkohene, Germany).

### 4.8. Isolation of the PM-Enriched Membrane Fraction from Roots of A. thaliana

The PM-enriched membrane preparations from *A. thaliana* WT and *Atflot1ko* knockout mutant plants, either exposed or not exposed to salt shock, were obtained using a water two-phase polymer system developed by Larsson et al. [49]. In variants with salt shock, NaCl at a final concentration of 100 mM was added to nutrient solutions 12 h prior membrane isolation. The membranes were isolated from roots at +4 °C, and all the solutions were precooled to this temperature before use. To prevent possible changes in the content of PM proteins which may occur during the slow cooling of plant material grown at +23 °C and then moved to a +4 °C cold room for membrane isolation, roots were separated from plants and then quickly immersed in a cooling solution (300 mM sucrose, 100 mM Tris-HCl, pH 8.0) prechilled to +4 °C. After 15 min incubation, the plant material was homogenized in a blender (12,000× *g*, 40 s) in the homogenization solution (300 mM sucrose, 100 mM Tris–HCl pH 8.0, 10 mM Na_2_-EDTA, 0.6% PVP K30 (polyvinylpyrrolidone), 5 mM K_2_S_2_O_5_, 5 mM DTT (dithiothreitol), and 1 mM PMSF (phenylmethylsulphonyl fluoride). The ratio of the homogenization medium to the plant material was 2:1 (*v*/*w*). The homogenate was filtered through two layers of cotton cloth and centrifuged at 10,000× *g* for 15 min. The resulting supernatant was then centrifuged at 186,000× *g* for 40 min in an ultracentrifuge (Beckman Coulter Optima L-90K, rotor type Ti 70). The microsome pellet was resuspended in the phase buffer (300 mM sucrose, 5 mM potassium phosphate buffer pH 7.8, 3 mM KCl, 1 mM DDT, and 0.1 mM Na_2_-EDTA). To separate membranes into subfractions, microsome suspension was mixed with a two-phase polymer system in a ratio of 1:3 (*v*/*v*). The polymer system was prepared by dissolving dextran T500 and PEG 3500 in a phase buffer, with the chosen components in a weight ratio of 1:1:10. The content of each polymer in the resulting mixture was 6.2 weight percent. The mixture of membrane vesicles with the two-phase polymer system was separated into two phases by centrifugation at 2500× *g* for 5 min (K-23 centrifuge, Janetzky, Germany). The upper PM-enriched phase was collected and diluted with the suspension medium (300 mM sucrose, 5 mM bis-tris propane-Mes, pH 7.2, 0.5 mM Na_2_-EDTA) at least four-fold. The diluted suspension was centrifuged at 186,000× *g* for 40 min in the Beckman ultracentrifuge. The resulting pellets were then resuspended in the suspension medium. The suspension of membrane vesicles was divided into 20 μL aliquots, frozen in liquid nitrogen, and stored at −70 °C for subsequent use.

### 4.9. Immunoblotting

The aliquots of *A. thaliana* PM preparations obtained from roots and leaves of WT and *Atflot1ko* knockout mutant plants were equally loaded (1 µg of total protein per lane) and separated on a 10% SDS-PAGE. The proteins from PAGE were transferred to a nitrocellulose membrane (NC) with a pore diameter of 0.45 μm (Schleicher & Schuell, Dassel, Germany) using a semi-dry device in accordance with the manufacturer’s instructions (Helicon, Moscow, Russia). The resulting NC membrane with bound proteins were stained using Ponceau S dye. For immunodetection of P-type H^+^-ATPase, rabbit polyclonal antibodies raised against a synthetic peptide containing a consensus sequence for H^+^-ATPase derived using a number of plant proteins, as well as H^+^-ATPases from other organisms (cat. # AS07 260, Agrisera, Vännäs, Sweden), were used as primary antibodies. Primary antibodies were used at a 1:1000 dilution in TBS-Tw-BSA (1xTBS, 0.2% Tween 20, 1% BSA, 0.01% NaN_3_), and secondary antibodies (goat anti-rabbit, horseradish peroxidase conjugate (HRP)) (Imtek, Ltd., Moscow, Russia) were used at a 1:2500 dilution in TBS/Tween 20-DM (1xTBS, 0.2% Tween 20, 5% Carnation non-fat dry milk (Nestle, Vevey, Switzerland)). Following incubation with primary and then secondary antibodies, the membrane was washed, and immunoreactive bands were visualized using an ECL kit (GE Healthcare, Piscataway, NJ, USA). A ChemiDoc XRS+ System gel documentation system (BioRad, Hercules, CA, USA) was used for visualization. Quantitative analysis of H^+^-ATPase content was carried out using the ImageJ program available at http://rsb.info.nih.gov/ij/ (accessed on 15 April 2020). For this analysis, for each lane, the areas containing two closely located major bands of H^+^-ATPase isoforms with molecular masses of approximately 100 and 115 kDa, respectively, and the space between them was selected.

### 4.10. Statistical Analysis

Statistical data processing was carried out using a one-way ANOVA analysis of variance (Microsoft Excel 2007). Data are presented as means and standard errors. Calculations were performed at a given significance level *p* ≤ 0.05.

## Figures and Tables

**Table 1:** Primers used in this study.

Name	Sequence 5′–3′	Experimental Purpose
AtPMA1_F1	AGG TGT GAT TTG GAT TTA CAG T	*PMA1/PMA2/AtFlot1/ACT2* gene expression studies using quantitative real-time RT-PCR (qRT-PCR)
AtPMA1_R1	CTG TTG TCA AAC AAG CTG G	
AtPMA2_F1	CTC AAC TTG TTT GAG AAC AAG ACG GC	
AtPMA2_R1	GGC TGT AAA CCG TGA AGT GTC C	
ACT2F	CTTGCACCAAGCAGCATGAA	
ACT2R	CCGATCCAGACACTGTACTTCCTT	
FlotRT_F	GGA ACC GAA GCA AGT GAC TC	
FlotRT_R2	TTC GCA TGG ATG TAT CTT CAA CCA C	
Flot1_9F18	CTAAATCATTTGGGCGAA	Confirmation of a T-DNA insert presence in the nucleotide sequence of the *AtFlot1* gene in *Atflot1ko* (SALK_205125C) mutant plants
Flot1_761R18	TTAGCTACATCAGCCTCC	

**Table 2:** The effects of NaCl on the H^+^-ATPase content in PM, endocytosis activity, and content of membrane-bound intracellular structures in *A. thaliana* plants of WT and the knockout mutant, *Atflot1ko*.

Genotype	WT	*Atflot1ko*
H^+^-ATPase content in PM	Increased	Decreased
Endocytosis activity (FM4-64 uptake)	No effect	No effect
GC and ER activity (cytoplasmic vesicles content)	Increased	Increased
GC content in cytoplasm	Increased	Decreased
MVB content in cytoplasm	Increased	No effect (in both control and saline conditions, MVB content is very low)
PMB content in the cells	Increased	Increased
PMBD content in vacuoles	Increased	No effect
H^+^-ATPase content in PM	Increased	Decreased
Endocytosis activity (FM4-64 uptake)	No effect	No effect
GC and ER activity (cytoplasmic vesicles content)	Increased	Increased
GC content in cytoplasm	Increased	Decreased
MVB content in cytoplasm	Increased	No effect (in both control and saline conditions, MVB content is very low)
PMB content in the cells	Increased	Increased
PMBD content in vacuoles	Increased	No effect

## Data Availability

Data are contained within the manuscript.
